# Cerenkov radiation-activated probes for deep cancer theranostics: a review

**DOI:** 10.7150/thno.75279

**Published:** 2022-10-24

**Authors:** Nian Liu, Xinhui Su, Xiaolian Sun

**Affiliations:** 1 PET Center, Department of Nuclear Medicine, the First Affiliated Hospital, Zhejiang University School of Medicine, Hangzhou 310003, China.; 2 State Key Laboratory of Natural Medicines, Key Laboratory of Drug Quality Control and Pharmacovigilance, Department of Pharmaceutical Analysis, China Pharmaceutical University, Nanjing 210009, China.

**Keywords:** Cerenkov radiation, probes, deep cancer, theranostics, radionuclides, X-ray radiation

## Abstract

Cerenkov radiation (CR) from radionuclides and megavoltage X-ray radiation can act as an *in situ* light source for deep cancer theranostics, overcoming the limitations of external light sources. Despite the blue-weighted emission and low quantum yield of CR, activatable probes-mediated CR can enhance the *in-vivo* diagnostic signals by Cerenkov resonance energy transfer and also can produce therapeutic effects by reactive species generation/drug release, greatly promoting the biomedical applications of CR. In this review, we describe the principles and sources of CR, construction of CR-activated probes and their application to tumor optical imaging and therapy. Finally, future prospects for the design and biomedical application of CR-activated probes are discussed.

## 1. Introduction

Light-based diagnosis and treatment methods have developed rapidly: examples include fluorescence imaging, optoacoustic imaging, photothermal therapy, and photodynamic therapy (PDT) 1-5. However, these methods are less effective for deep tumors because of the rapid attenuation of light as it passes through tissue 6. To overcome this limitation, Cerenkov radiation (CR) from radionuclides and megavoltage X-ray radiation can serve as an *in situ* excitation source within deep tissues 7. Blue-weighted CR occurs when a charged particle travels faster than the speed of light in a given medium 8, 9. Improvements in optical imaging technology and the increasing sensitivity of CCD cameras allow the detection of blue-weighted CR photons for functional molecular imaging 10-11. Preclinical and clinical explorations of CR imaging have demonstrated its potential for high throughput, high sensitivity, and rapid imaging 12-17. Nevertheless, current CR-based imaging lacks the necessary depth because the blue-weighted CR photons do not penetrate deep enough to image certain tumors 10, 18.

Activatable probes-mediated CR provides a way to enhance the diagnostic signals or produce therapeutic effects 19, 20. These optical probes participate in Cerenkov resonance energy transfer (CRET) to red-shift emission into the near-infrared (NIR) region or red region of the visible spectrum, enabling sensitive detection from deeper inside the tissue. Besides, some activatable probes can produce therapeutic effects at greater depths: for example, ultraviolet (UV)-responsive probes can be activated by CR to produce therapeutic reactive species or to release drugs, referred to as CR-induced therapy (CRIT) 21. The combination of activatable probes and CR expands the biomedical applications of CR.

In this review, we summarize CR-activated probes that have been reported to enhance optical imaging and therapy. CR efficiently triggers these optical probes with red-shifted emission, which include quantum dots, gold nanoclusters, lanthanide-based downconversion probes, persistent luminescence nanoparticles (PLNPs), and organic dye-associated NPs. The CR-induced PDT, photoimmunotherapy, drug release and even combinations of these therapies are also discussed in detail.

## 2. Cerenkov radiation

### 2.1 Principles of CR

CR is generated through the interaction of charged particles and matter 9. It can be produced from radionuclides, megavoltage X-ray radiation, cosmic events and nuclear reactors; clinical facilities may have access to radionuclides and linear accelerators 19. The radionuclides release α, β, or γ particles during the decay, and the subatomic particles are charged when polarizing the medium. A similar process occurs in megavoltage X-ray radiation, except that the kinetic energy of the resulting electrons is 2-3 orders of magnitude greater when they pass through tissue 15.

**Figure 1:**
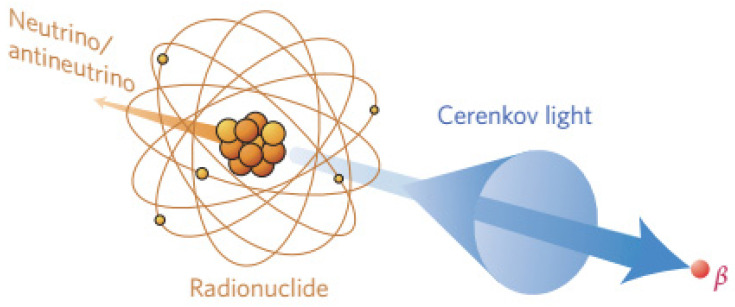
CR is produced by the medium through which charged particles propagate. Reproduced with permission 19. Copyright 2017, Nature Publishing Group.

When a charged particle travels faster than the speed of light through a dielectric medium, the surrounding molecules in the medium become locally polarized. After the particles pass through the medium, the molecules return to their ground state and release blue-weighted light called CR (**Figure 1**) 15, 19. CR intensity is related to the velocity and energy of the charged particles (υ), as well as the refractive index of the medium (c). The threshold of input energy needed to generate CR can be described as [19]:



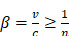



Additionally, the photon flux of CR at a given frequency can be calculated using the Frank-Tamm equation:



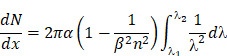



which demonstrates that CR is blue-weighted and decreases with 1/λ^2^ from the UV to infrared regions 19.

### 2.2 Sources of CR

#### 2.2.1 Radionuclides

Radionuclides undergo decay through capture of positrons (β^+^) or electrons (β^-^), followed by an isomeric transition. CR arises when charged particles travel faster through the surrounding medium than the phase velocity of light 22. **Table 1** lists radioisotopes that emit energy over the CR threshold, such that they induce CR emission 23. Positron emitters in clinical use of positron emission tomography (PET) include ^18^F, ^64^Cu, ^68^Ga, ^74^As, ^89^Zr, and ^124^I. Electron emitters in clinical use include ^32^P, ^90^Y, ^131^I, ^177^Lu, and ^198^Au, which can also be used for radiotherapy of cancer.

The radioactivity of each radionuclide strongly influences the intensity of the produced CR. In CR-based imaging, radionuclides with a relatively short half-life, such as ^18^F and^ 64^Cu, are preferred for building theranostic nanomaterials. In CRIT, radionuclides with a long half-life, such as ^90^Y, ^131^I and ^177^Lu, are preferred because they can generate CR in the long term for prolonged treatment, and they can generate ionizing radiation that provides radiotherapy.

#### 2.2.2 Megavoltage X-ray radiation

**Figure 2:**
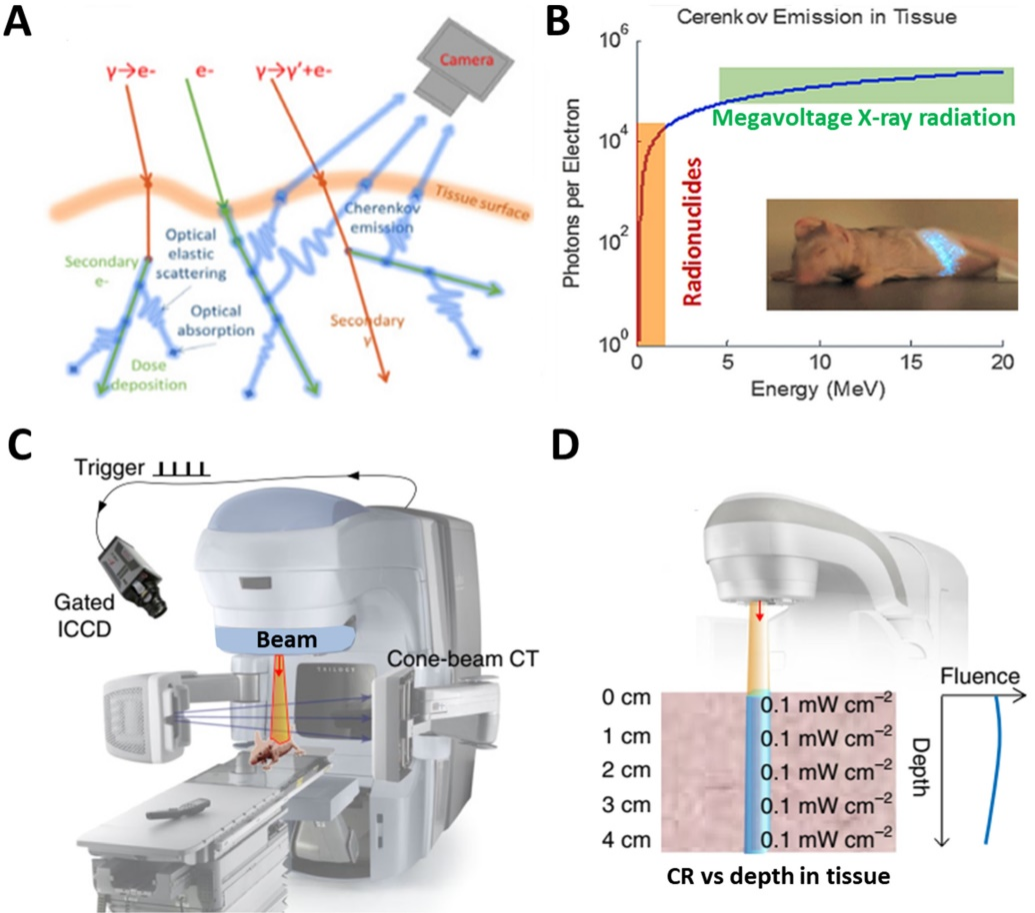
** (A)** X-rays induce CR through soft collisions of electrons. **(B)** Numbers of Cerenkov photons per electron generated from radionuclides or a linear accelerator. Reproduced with permission 7. Copyright 2021, Springer-Verlag Wien. **(C)** Experimental setup for scanning imaging based on Cerenkov-excited luminescence. **(D)** Fluence of X-ray induced CR from different depths. Reproduced with permission 27. Copyright 2018, Nature Publishing Group.

The emission of CR needs the irradiation energy of X-rays to meet the in the high kilovoltage or megavoltage range while only 1% of secondary electrons can be delivered 25, 26. Using X-rays to generate CR and in turn induce luminescence allows imaging at sub-millimeter resolution with nanomolar sensitivity, such as during real-time monitoring during radiotherapy *in vivo* 25, 26. When X-rays pass through tissues, soft collisions during energy deposition lead to de-excitation of primary or secondary electrons, generating Cerenkov emission (**Figure 2**A) 7. Megavoltage X-ray radiation is an efficient CR source because the X-ray beams contain far more Cerenkov photons per electron than what traditional radionuclides generate **(Figure 2**B) 7. An experimental scanning imaging system to detect CR-excited luminescence is shown in **Figure 2**C. The system includes an accelerator beam and an intensified charge-coupled device. The CR shows minimal fluence loss as it propagates from deep within tissue to the detector (**Figure 2**D) 27.

## 3. Construction of CR-activated probes

**Figure 3:**
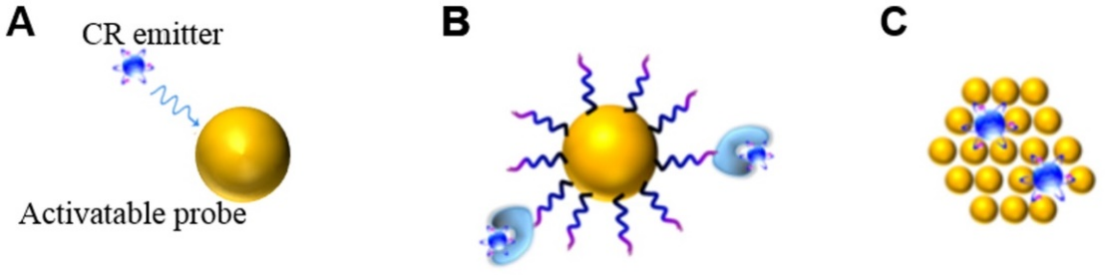
Interactions between a CR emitter and activatable probe in the case of **(A)** an unbound Cerenkov emitter, **(B)** chelator-bound CR emitter, or **(C)** intrinsic radiolabelling. Reproduced with permission 28. Copyright 2015, American Chemical Society.

CR can be combined in space and time with activatable probes in two ways. One way is the unbound CR emitter with probes. Megavoltage X-ray radiation or clinical radiopharmaceuticals such as ^18^F-fluorodeoxyglucose (^18^F-FDG) can be used, which requires co-localizing the CR and probes in time and space *in vivo*. The other way is to use probes already labeled with radionuclides, mainly through (1) extrinsic chelation or (2) intrinsic radiolabelling (**Figure 3**) 28. The most appropriate radiolabeling chelator depends on the coordination chemistry of the radiometal ion and the surface chemistry of the probe 29. Commonly used chelators include diethylene triamine pentaacetic acid (DTPA), desferrioxamine (Df), 1,4,7,10-tetraazacyclododecane-1,4,7,10-tetraacetic acid (DOTA), and 1,4,7-triazacyclononane-1,4,7-triacetic acid (NOTA). The chelation should be carefully designed in order to avoid altering the probe's pharmacokinetics. This approach carries risk of radionuclide detachment, leading to nonspecific biodistribution that leads to “noise” in the images. Intrinsic radiolabeling means incorporating radionuclides directly into nanoprobes, through such methods as “hot-plus-cold precursors”, cation exchange, and specific trapping 29. For example, radioactive and non-radioactive precursor components can be mixed during NPs synthesis, or the radiochemical can be generated after nanoformulations through surface elemental exchange or radionuclide deposition. Intrinsic radiolabeling ensures a highly stable label that does not alter the probes' intrinsic behavior.

## 4. Probes for imaging based on CRET

A series of optical probes have been developed to minimize signal attenuation as it passes through tissue, yet tissue autofluorescence still generates sufficient background to severely degrade image quality 30. CR can act as an *in situ* light source to activate fluorescent probes with large Stokes shifts. In this way, CR overcomes the limited penetration depth of external light, and it avoids tissue autofluorescence. At the same time, the excited fluorescent probe shifts blue-weighted CR to the NIR region or the red visible region, resulting in deeper tissue penetration. Below, we summarize different types of CR-excited fluorescent probes for optical imaging of tumors (**Table 2**).

### 4.1 Quantum dots

Quantum dots (QDs) are nanoscale crystalline clusters (1-10 nm) with high quantum yields, large spectral shifts, and tunable emission colors. They have attracted substantial attention for *in vivo* fluorescence imaging 31, 32. They absorb broadly throughout the UV-visible range, overlapping with blue-weighted CR. ^64^Cu and ^18^F have been used to stimulate Qtracker705 QDs, and high CRET from the enhanced photons in the >590 nm filter was achieved during imaging of phantoms and tissues *in vivo* 33. Na^131^I has been used as an unbound CR emitter to activate three CdSe/ZnS QDs with emission peaks of 665, 705, and 800 nm, thereby achieving multiplexed optical imaging 34. In an *in vivo* study, ^89^Zr-labeled deferoxamine-trastuzumab actively targeted BT-474 tumors, and intravenously injected cRGD-QD605 accumulated in the tumor region, allowing tumors to be imaged through secondary Cerenkov-induced fluorescence 35. Nevertheless, that work found that the radionuclides did not completely co-localize with the QDs. To improve co-localization, CdSeTe, CdSe, and CdZnS were modified with ^89^Zr-labeled chelator, and the resulting self-illuminating systems enabled good mapping of sentinel lymph nodes and prostate cancer 36.

**Figure 4:**
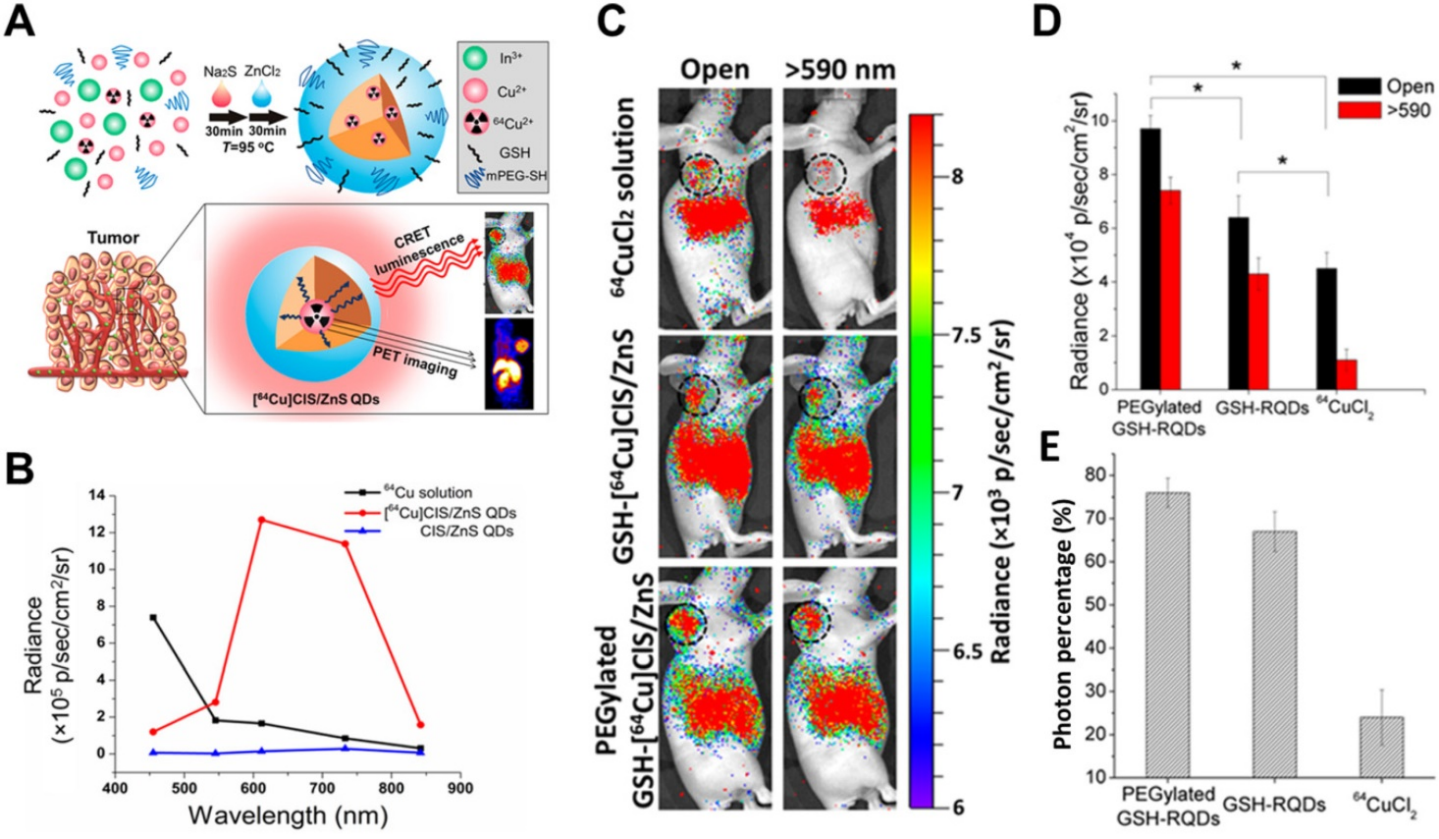
** (A)** Schematic of chelator-free synthesis of radiolabeled QDs and application to PET and CRET imaging. **(B)** Photon flux of different samples obtained with different filters. **(C)** CRET images of U87MG tumor-bearing mice after 6 h of different treatments. **(D,E)** Total photon fluxes and red filter ratios in the tumor region. Reproduced with permission 38. Copyright 2015, American Chemical Society.

To avoid radionuclide detachment, we developed two chelator-free methods to generate ^64^Cu-labeled QDs 37, 38. One is to introduce ^64^Cu into QDs by cation exchange 37, and the other is to use ^64^CuCl_2_ as a precursor when synthesizing CuInS/ZnS (**Figure 4**) 38. These two methods lead to high radiostability. The two types of ^64^Cu-labeled QDs exhibited higher tumor uptake and signal-to-background ratio in CRET imaging *in vivo*. CR was induced by X-rays to excite PdSe QDs, which emitted in the short-wave infrared region and CRET allowed sensitive depth imaging 39.

### 4.2 Lanthanide-based downconversion probes

Lanthanide-based downconversion probes possess good characteristics for optical imaging, such as narrow emission bandwidths, large Stokes shifts, and good photostability 40, 41. They are usually synthesized by doping Tb^3+^, Eu^3+^, and Dy^3+^ to achieve the traditional Stokes luminescence, and they are activated by UV light to generate visible-NIR emission 42. Therefore, lanthanide-based downconversion probes may be good CRET mediators to harness CR energy. Below we summarize the two groups of probes, lanthanide nanoparticles (NPs) and lanthanide complexes.

**Figure 5:**
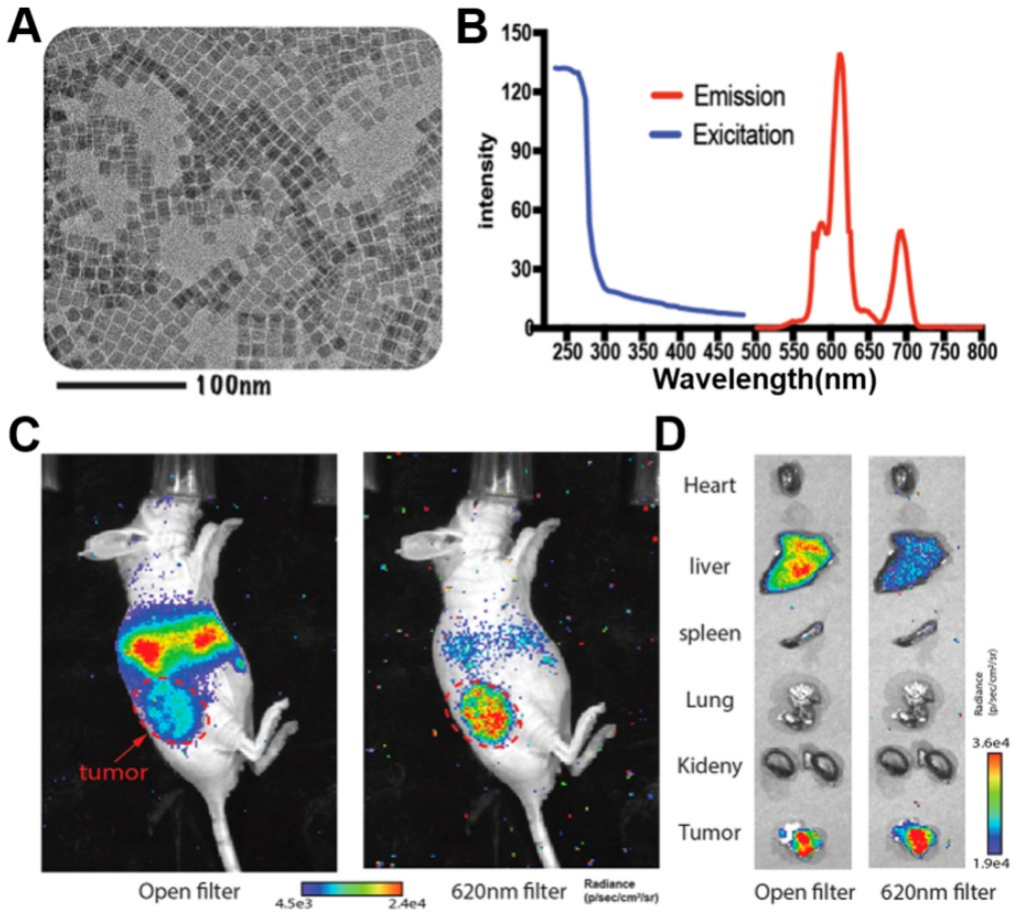
** (A, B)** TEM image, excitation and emission spectra of ultrasmall Eu_2_O_3_ NPs. **(C, D)** CRET images of CT26 tumor-bearing mice and *ex vivo* organ imaging after 48h's injection of ^89^Zr- Eu_2_O_3_. Red arrow, tumor area. Reproduced with permission 50. Copyright 2021, American Chemical Society.

Lanthanide NPs with hard-core structures are usually synthesized by high-temperature chemical methods. Eu^3+^-doped Ba_0.55_Y_0.3_F_2_ nanophosphors with emission peaks at 597, 615, 692 nm have been prepared by a solvent-thermal method. Through CRET, excitation by ^18^F-FDG enhanced nanophosphor radiance at 700 nm for phantom and animal imaging 43. Eu^3+^-doped Y_2_O_3_ NPs have also used as CR mediators for CRET imaging 44; CR, rather than γ rays from ^68^Ga, was confirmed to enhance luminescence 44. CR from ^18^F-FDG as well as β and γ radiation efficiently activated Eu^3+^-doped Gd_2_O_3_ NPs, enhancing optical imaging and intraoperatively guiding tumor surgery 45. To improve the efficiency of photon leap in lanthanide NPs, Eu_2_O_3_ NPs, which have multiple absorption peaks in the UV range, have been proposed to show more efficient photon leap in lanthanide NPs after activation by ionizing radiation including CR, β, and γ scintillation. This reflects the fact that β interactions significantly contribute to activation of Eu_2_O_3_ 46-49. Indeed, this approach allowed radiopharmaceutical-excited fluorescence imaging of phantoms as well as Bcap-37, U87MG, and 4T1-luc2 tumor models *in vivo* with high signal-to-background ratio. Also, Ultra-small ^89^Zr-labelled Eu_2_O_3_ NPs have been developed for enhanced Cerenkov imaging, allowing clear imaging of lymph nodes and tumors (**Figure 5**) 50.

Other lanthanide complexes have been designed to be excited *in situ* by radionuclides. ^18^F- or ^89^Zr-excited terbium(III) complexes of a macrocyclic polyaminocarboxylate ligand achieved a detection limit of 2.5 nmol in CRET luminescence 51. Peptide-functionalized Tb(III) and Eu(III) complexes have been constructed for radiopharmaceutical-activated imaging* in vivo*. Combining these two probes with ^18^F-FDG led to emission at 570 and 620 nm, allowing multiplexed optical imaging of tumors 52.

### 4.3 Gold nanoclusters

**Figure 6:**
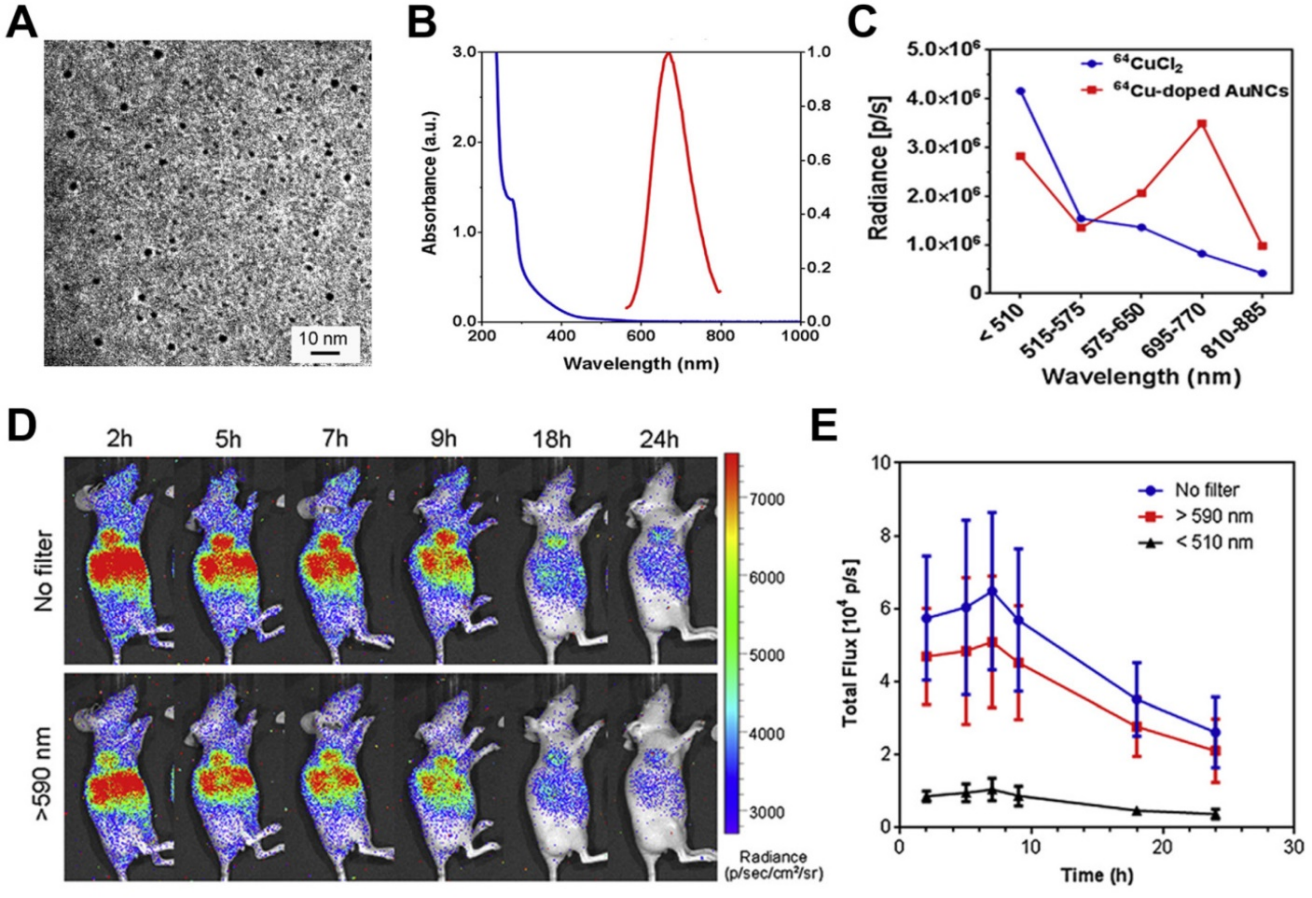
** (A)** TEM image of Cu-doped AuNCs (1%Cu). **(B)** The absorption and emission spectrum of Cu-doped AuNCs (1% Cu). **(C)** Intensity changes of ^64^CuCl_2_ and ^64^Cu-doped AuNCs at different optical filter sets. **(D, E)** Representative CRET images and photon flux of ^64^Cu-doped AuNCs on the U87MG tumor model. Reproduced with permission 54. Copyright 2014, Elsevier.

Gold nanoclusters (AuNCs) have been extensively developed as fluorescent probes for biomedical applications owing to their advantages of high compatibility, strong fluorescence, superior photostability, and excellent water solubility 53. AuNCs are usually biomineralized with naturally functional macromolecules, which provide good colloidal stability and facilitate surface functionalization. AuNCs can fluoresce across a broad region extending from visible to near-infrared regions with a large Stokes shift. ^64^Cu-doped AuNCs with a diameter of 2.54 nm have been synthesized using an approach directed by human serum albumin, leading to AuNCs with an absorption peak at 280 nm and an emission peak at 667 nm (**Figure 6**A, B) 54. During imaging of phantoms and a U87MG tumor model, ^64^Cu-doped AuNCs showed 4.3-fold greater optical intensity than free ^64^CuCl_2_ with an optical filter of 695-770 nm but lower intensity below 510 nm, demonstrating CRET from ^64^Cu to AuNCs (**Figure 6**C, D, E).

To verify the mechanism of efficient radioisotope energy transfer by AuNCs, they were excited using ^18^F-FDG, ^90^Y, or ^99m^Tc (γ-emitter). Activation of ^99m^Tc did not induce optical signal, while activation of ^18^F-FDG and ^90^Y enhanced optical emission 55. The fact that the signal enhancement was not proportional to CR implies that AuNCs were excited by CR and direct Coulombic interaction 55.

### 4.4 PLNPs

**Figure 7:**
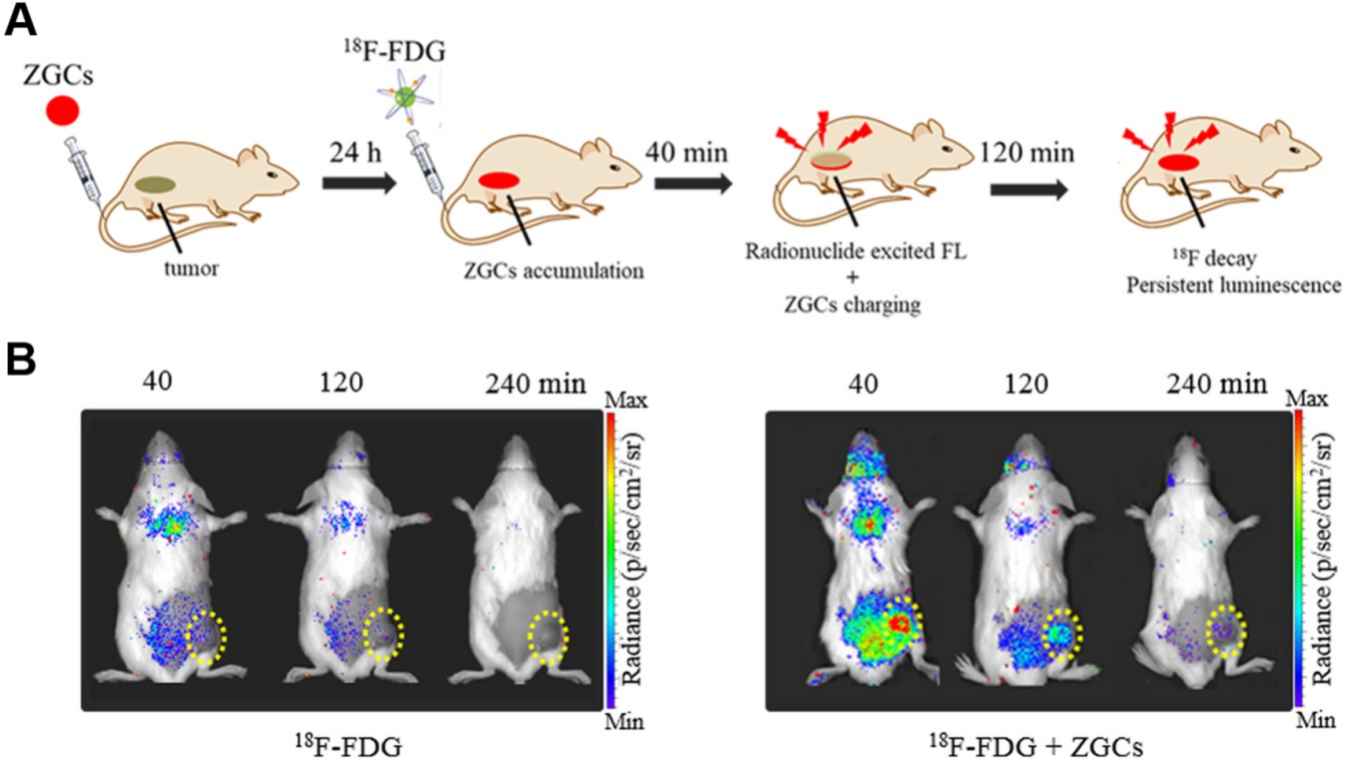
** (A)** An illustration of *in vivo* ^18^F-FDG activated persistent luminescence imaging of ZGCs. **(B)** Representative luminescence images of 4T1 tumor-bearing mice after administration of only 200 μCi ^18^F-FDG or 200 μg ZGCs injection before 24 h and following with 200 µCi^ 18^F-FDG. Reproduced with permission 61. Copyright 2020, Wiley-VCH.

PLNPs maintain their luminescence after excitation has stopped, so they have been exploited for autofluorescence-free optical imaging with different excitation sources 56-58. Cr^3+^-doped zinc gallates (ZGCs) are NIR-emitting PLNPs commonly used for tumor diagnosis, with three main excitation bands with peaks at 260, 465, and 570 nm 59, 60. For the first time, we report that both CR and γ radiation from ^18^F-FDG can efficiently activate ZGCs to emit in the NIR region and persistently release photons for long-term imaging (**Figure 7**A) 61. In fact, ^18^F-FDG excited ZGCs in 4T1 tumors in mice, maintaining luminescence for longer than 3 h (**Figure 7**B). Multiple injections of ^18^F-FDG also allowed for long-term real-time observation of tumor status, which may facilitate image-guided surgery in the future.

### 4.5 Organic dye or associated NPs

**Figure 8:**
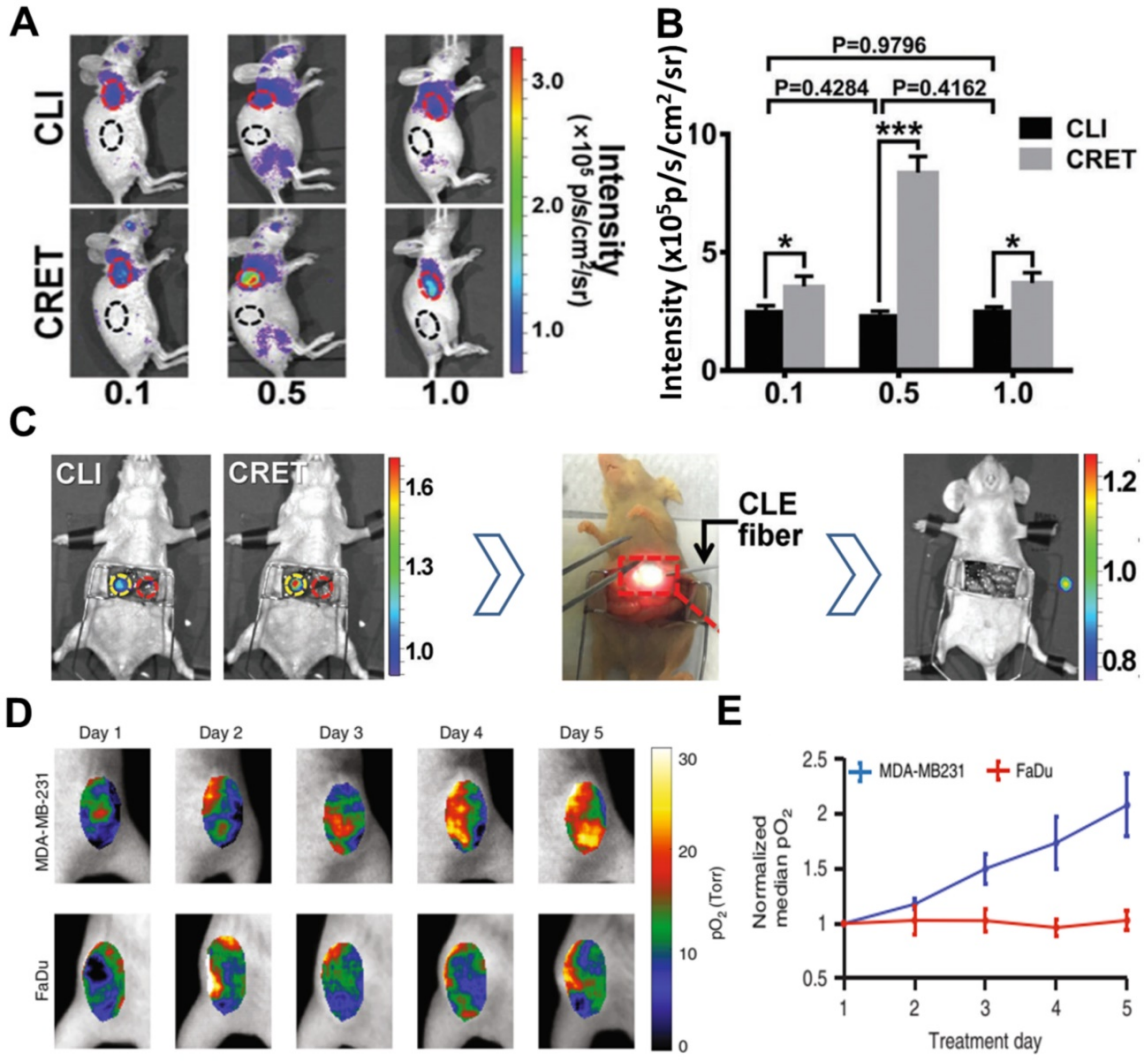
** (A)** CR and CRET imaging of subcutaneous 4T1 tumor model after injection of 300µCi ^18^F-FDG (CRET group was treated with different doses of fluorescein). **(B)** The signal intensities and TNR of CLI and CRET. **(C)** Intraoperative CRI and CRET imaging of orthotropic HCC tumor model, and the process of image-guided surgery. Reproduced with permission 64. Copyright 2019, Wiley-VCH. **(D, E)** pO2 images and the corresponding pO2 changes during each day of radiation (6MV X-ray beam with 5 Gy/fraction), PtG4 was iv injected 24h before the first treatment. Reproduced with permission 73. Copyright 2019, Nature Publishing Group.

Inorganic fluorescent probes have excellent optical properties and can take full advantage of CR to undergo highly efficient CRET with large Stokes shifts. However, their potential toxicity and non-biodegradability have prevented their extensive development for clinical use. Organic compounds, in contrast, show good biodegradability and low toxicity 62. For example, fluorescein sodium is clinically used to image retinal blood vessels; it absorbs strongly at 465-490 nm and emits at 520-530 nm 63, 64. Despite its short Stokes shift, fluorescein sodium can be activated by ^18^F-FDG or ^11^C-CHO to show efficient CRET, as demonstrated in a subcutaneous 4T1 tumor model and orthotopic hepatocellular carcinoma tumor model (**Figure 8**A, B,C) 64. In that study, tumors were precisely and completely resected through intraoperative CRET and confocal laser endomicroscopy imaging 64.

Various fluorophores have been used to convert CR to NIR emission. Excitation of a mixture of fluorophores (fluorescein, rhodamine 6G, rhodamine 101, gresyl violet, cyanine 5, and indocyanine green) with ^90^YCl_3_ led to a CRET process, followed by several rounds of Förster resonance energy transfer (FRET), culminating in emission at 710 nm 65, 66. All these fluorophores overlapped well in spectra, emitting twice as many photons as the radionuclide on its own. Featuring larger Stokes shifts, water-soluble phthalocyanine-pyranine conjugates have been developed with absorption at 250-450 nm and emission at 700 nm. They can be activated by ^18^F-FDG, leading to strong CRET using a filter of 710 nm as well as a photodynamic effect 67. Interestingly, ^18^F-labeled naphthofluorescein derivatives have been developed that show selective bandwidth quenching under alkaline conditions but full-spectrum CR under acidic conditions 68.

Comparison of different CR-excited fluorophores induced by X-rays 69-72 suggests that aluminum phthalocyanine may be better for molecular luminescence 69, while platinum II G4 (PtG4), with absorption peaks at 435 and 624 nm and an emission peak at 780 nm, may be better for *in vivo* sensing 70. PtG4 and X-ray induced CR have been used to detect spatial distribution of oxygen in tumors based on phosphorescence lifetimes 27, 73. This approach revealed much higher partial pressure of oxygen (pO_2_) in MDA-MB-231 tumors than in FaDu tumors in animal models, suggesting that MDA-MB-231 tumors are more susceptible to radiotherapy (**Figure 8**D, E).

Organic dye-based NPs can enhance the accumulation of contrast agents in tumors while preserving the optical property of those agents. [Ir(pq)_2_(bpy)]Cl has been encapsulated into liposomes to formulate Ir@liposome, then irradiated with ^18^F-FDG, leading to imaging of deep tissue with a high signal-to-noise ratio 74. Pluronic F127 silica NPs doped with five dyes have been constructed to absorb strongly in the visible range, leading to the efficient shift of CR towards NIR emission with a quantum yield of 0.12 75. The highly efficient energy transfer allowed the NPs to be detected from muscle as thick as 1.0 cm.

## 5. Probes for CR-induced therapy

CRIT refers to therapeutic effects that occur when CR-activated probes directly or indirectly produce reactive molecules that kill tumor cells. The efficiency of CRIT depends on delivery of adequate CR to target tissue, and proper stimulation of the probe. Therefore, the CR emitter and activatable probe must be selected carefully. Of course, effective tumor suppression also requires co-localization of emitter and probe. In addition, toxic effects that arise when the two inevitably co-localize in normal tissues must also be considered.

Below we provide an overview of probe-based CRIT, including CR-induced PDT, CR-induced photoimmunotherapy, CR-triggered drug release, and combination therapy (**Table 3**).

### 5.1. CR-induced PDT

PDT has been widely used to treat many solid tumors because of its non-invasiveness and dual selectivity 76. In PDT, external light activates photosensitizers to generate reactive oxygen species (ROS), which damage cancer cells. A major drawback is that PDT requires direct irradiation of tissue with visible or even UV light in order to stimulate photosensitizers. This limits penetration depth, making the approach ineffective against large or deep-seated tumors 6, 77. Blue-weighted CR can overcome this problem by acting as an *in situ* light source to activate photosensitizers continuously 21.

**Figure 9:**
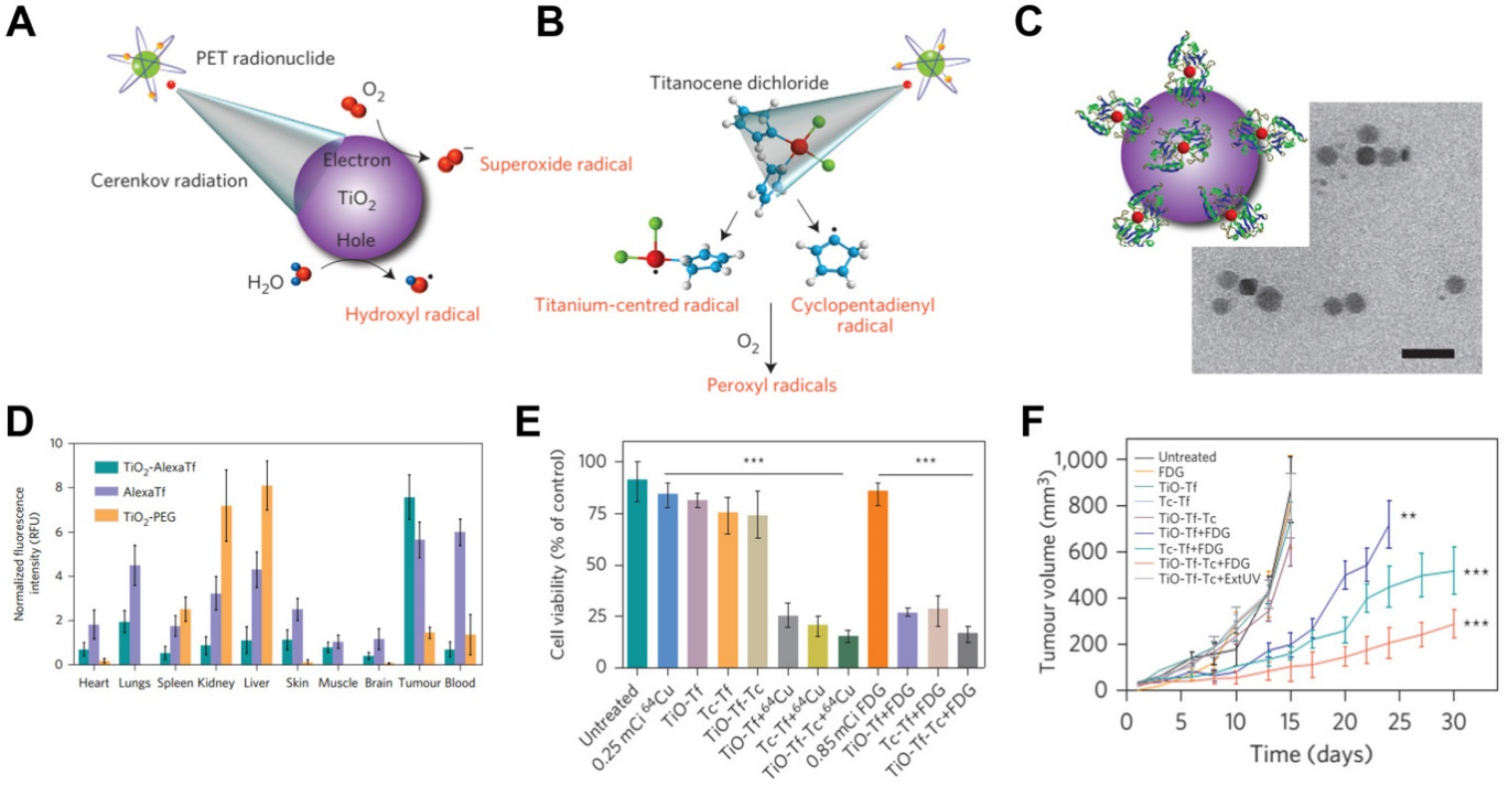
** (A, B)** An illumination of CR-induced PDT during the CR-excited TiO_2_ NPs and Tc. **(C)** TEM image of TiO_2_-Tf-Tc. **(D)** *In vivo* biodistribution of TiO_2_-PEG and TiO_2_-AlexaTf at 24h postinjection in HT1080 tumor-bearing mice. **(E)** Cell-viability assays on HT1080 cells with various treatments. **(F)** Tumor volumes of HT1080 tumor-bearing mice with various treatments. Reproduced with permission 79. Copyright 2015, Nature Publishing Group.

For example, TiO_2_ NPs act as photocatalysts that can be regenerated to efficiently absorb UV light at 275-390 nm and to produce cytotoxic superoxide radicals and hydroxyl 78. A TiO_2_-based CRIT-nanoplatform has been achieved for deep PDT, in which ^18^F-FDG excites TiO_2_ to continuously generate OH^-^ by electron-hole pair generation (**Figure 9**A) 79. Of course, it is worth noting that β and γ scintillation produced from radionuclides also play a role in TiO_2_'s activation 49. Intratumoral administration of TiO_2_-PEG and ^64^Cu led to remarkable tumor regression within 3 days and complete inhibition within 30 days. In another approach, apo-transferrin, a ligand for overexpressed Tf receptors, and titanocene, a photogenerator of peroxyl radicals, were integrated into TiO_2_ (TiO_2_-Tf-Tc) to enhance CRIT with an intravenous platform (**Figure 9**B,C,E). This system accumulated in tumors to a greater extent than TiO_2_-PEG within 24 h, based on *ex vivo* fluorescence imaging (**Figure 9**D). Intravenous injection of TiO_2_-Tf-Tc followed by ^18^F-FDG at 24 h later significantly inhibited tumor growth (**Figure 9**E,F). This work paves the way to treating deep-seated tumors using CR-induced PDT.

To compensate for low uptake of ^18^F-FDG in bone, ^89^Zr-labeled TiO_2_-Tf has been developed to target bone marrow with high selectivity, leading to accumulation of 70% of injected radioactivity there. This accumulation allowed continuous ROS production that completely eliminated tumors from a multiple myeloma model and that doubled survival time 80. Tf-mediated transport of titanocene into various types of tumor has been improved by using high-affinity VLA-4-bonded LLP2A conjugated phospholipid micelles or human serum albumin NPs 81. Activation of the titanocene *in situ* by intravenously injected ^18^F-FDG inhibited breast cancer metastasis and growth of disseminated multiple myeloma. ^68^Ga-bovine serum albumin has been used instead of ^18^F-FDG to improve PDT efficiency with CR-excited TiO_2_, leading to strong tumor inhibition *in vitro* and *in vivo* 82.

**Figure 10:**
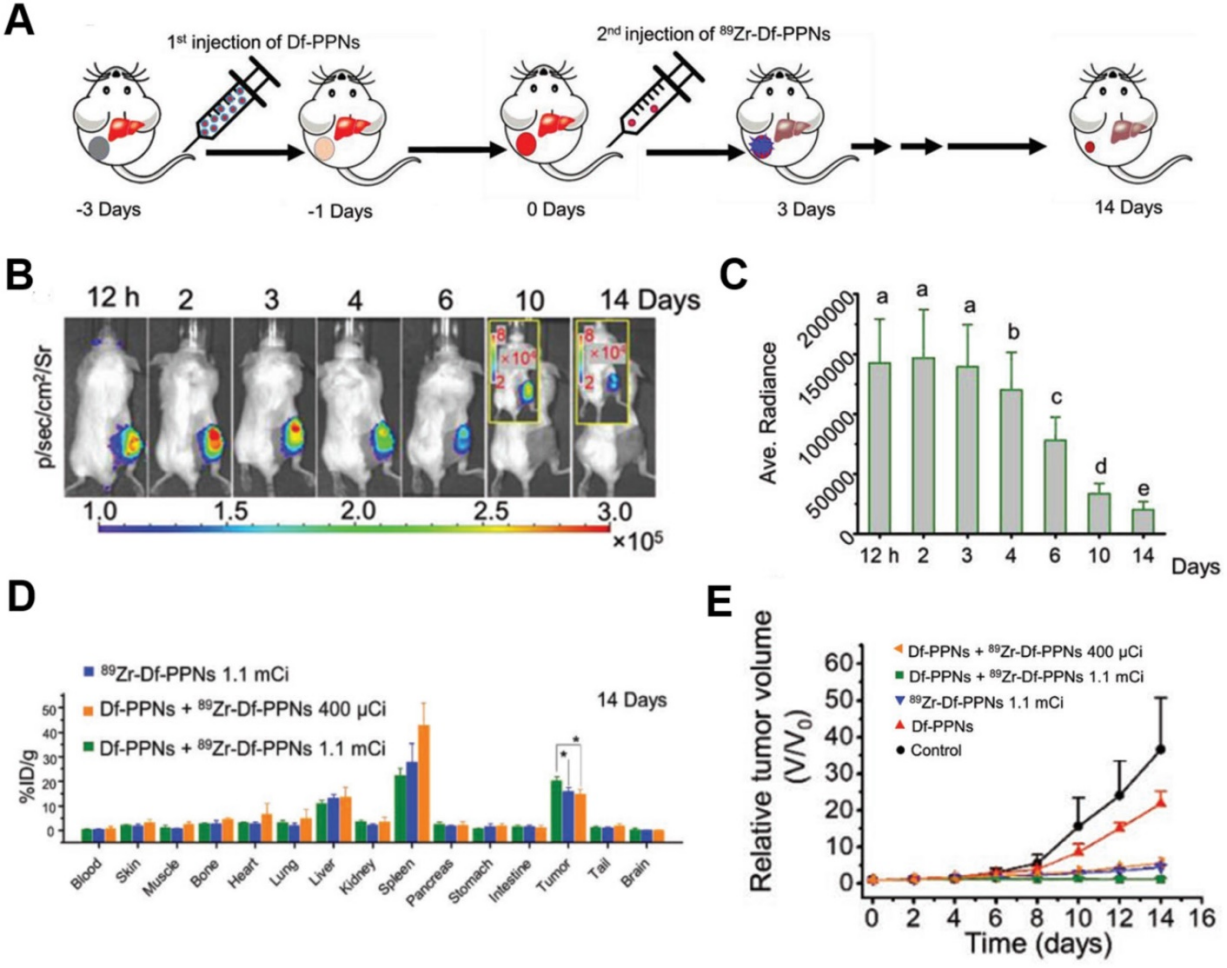
** (A)** Schematic of the “missile-detonation” strategy. **(B,C)** Representative CR-excited optical images and the corresponding photon fluxes in a 4T1 tumor model on different days after treatment. **(D)** Biodistribution of ^89^Zr-Df-PPNs at 14 days after injection, based on measurement of radioactivity in each organ. **(E)** Relative volume of 4T1 tumors in mice after various treatments. Reproduced with permission 85. Copyright 2019, Wiley-VCH.

CR-induced PDT has been achieved using the photosensitizer dyes, such as chlorin e6 and tetrakis(4-carboxyphenyl)porphyrin (TCPP) 83-85. Hollow mesoporous silica NPs were loaded with ^89^Zr-labeled chlorin e6, and the ^89^Zr provided CR to activate chlorin e6, which generated tumor-inhibiting ROS 83. Given that intravenously injected theranostic agents do not strongly accumulate in tumors, ^89^Zr-labeled magnetic NPs with a modified porphyrin surface were constructed to enable accumulation of the NPs in tumors (15% ID/g) after application of a magnetic field; the accumulation was five times higher than without the magnetic field 84. TCPP-loaded magnetic NPs significantly inhibited tumor growth through CR-induced PDT during two weeks. While promising, this strategy led to substantial NPs accumulation also in healthy organs, suggesting the potential for adverse effects. To enhance targeting in CRIT, a “missile-detonation” strategy has been developed: high-dose p-SCN-Bn-deferoxamine-porphyrin-PEG nanocomplex (Df-PPN) was administered as a CR energy receiver, and it passively targeted tumors; then, at the optimal time, low-dose ^89^Zr-labeled Df-PPN was injected as a CR emitter (**Figure 10**A) 85. This strategy effectively mitigated toxic side effects of CRIT due to the relatively low accumulation of PPN in normal tissues. The nanocomplex persisted in the tumor in the long term, based on CR-excited fluorescence imaging (**Figure 10**B,C,D), and it drove continuous ROS production that significantly suppressed tumor growth (**Figure 10**E).

### 5.2 CR-induced photoimmunotherapy

NIR photoimmunotherapy is a newly targeted cancer treatment that uses antibody-photoabsorbers (IRDye700DX) to actively bind to cancer cells and then induce the selective immunogenic cell death under NIR light irradiation, resulting in the activation of anti-cancer immune system locally in the tumor microenvironment 86, 87. However, NIR laser light cannot penetrate deep in tissue, but the CR emitter ^18^F-FDG can be delivered deep into tissue to deliver radiation at the absorbance peak of IRDye700DX at 350 nm, allowing photoimmunotherapy of deep-seated tumors 88. The CR-induced photoimmunotherapy in that study inhibited tumor growth to some degree, but the effect was not consistent during the observation period. This may be because the lower CR energy stimulated a weak immune response. Megavoltage X-ray radiation may be a better CR source for activation of IRDye700DX.

### 5.3 CR-triggered drug release

**Figure 11:**
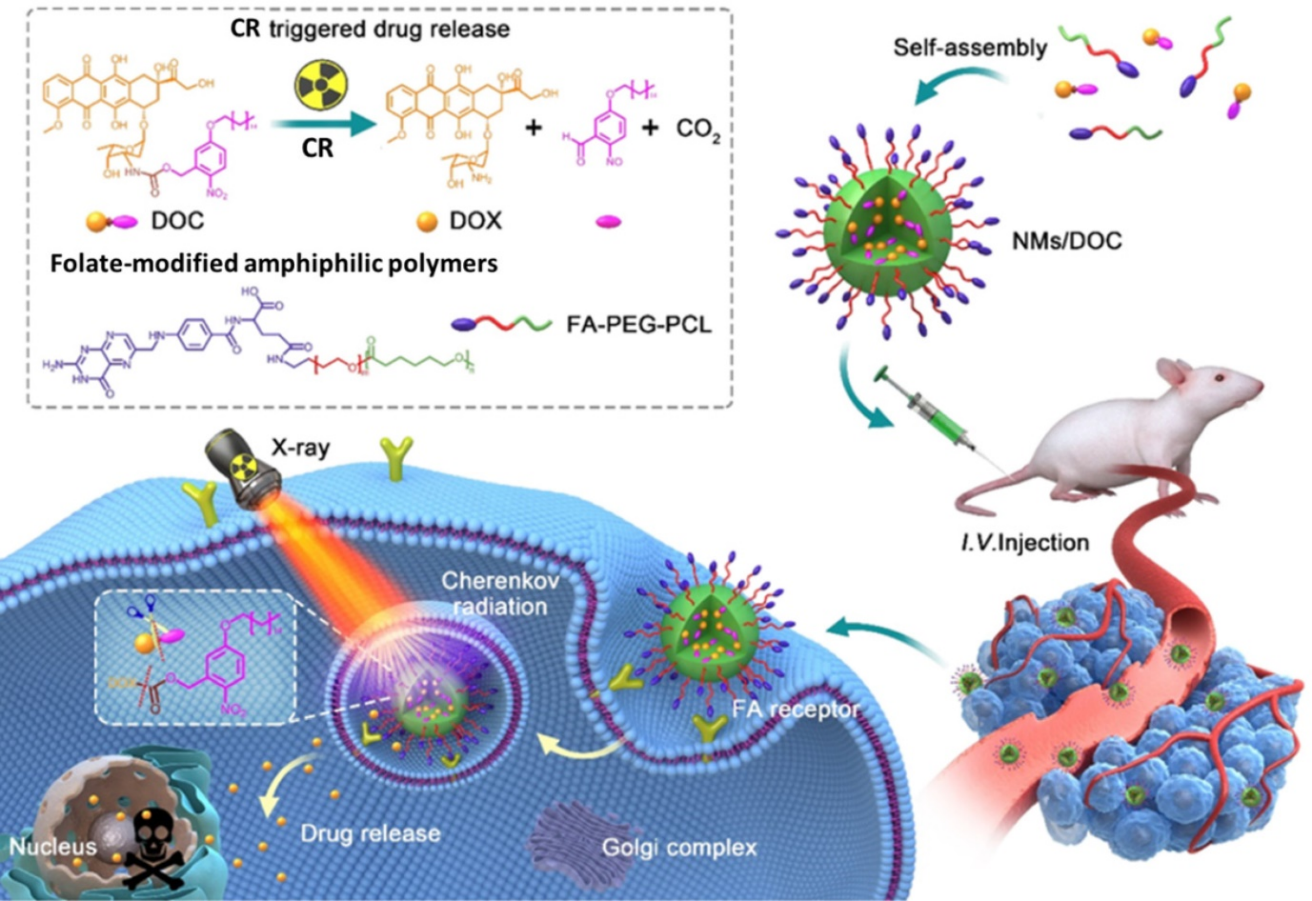
The CR-responsive folate-modified DOX/nanomicelles for folate cellular targeting and nuclear localization toxicity. Reproduced with permission 92. Copyright 2020, American Chemical Society.

Although chemotherapeutics can be effective against cancer, delivering them to target tissue without causing toxicity in normal tissue is challenging 89. Therefore, drug delivery systems have been developed to release drugs at specific sites in response to certain stimuli, especially light 89, 90. However, the limited penetration of light through tissue means that external light sources cannot efficiently trigger drug release in deep tissue. Using CR as *in situ* light can overcome this shortcoming. UV-responsive drug delivery systems may be most appropriate for CR-activated drug release. For example, phenacyl bis-azide crosslinkers make dextran-based hydrogels photosensitive, and the co-loaded doxorubicin (DOX), BSA, and IgG are efficiently released after UV light irradiation 91. In another approach, DOX has been caged using a photocleavable o-nitrobenzyl ester derivative and then encapsulated into nanomicelles for CR-triggered drug release and chemotherapy (**Figure 11**) 92. Irradiation with X-rays induced CR, which converted the hydrophobic DOC into hydrophilic DOX, which was rapidly released and killed cancer cells. This strategy may pave the way to a new chemoradiotherapy with minimal systemic side effects.

### 5.4 Combination therapy

**Figure 12:**
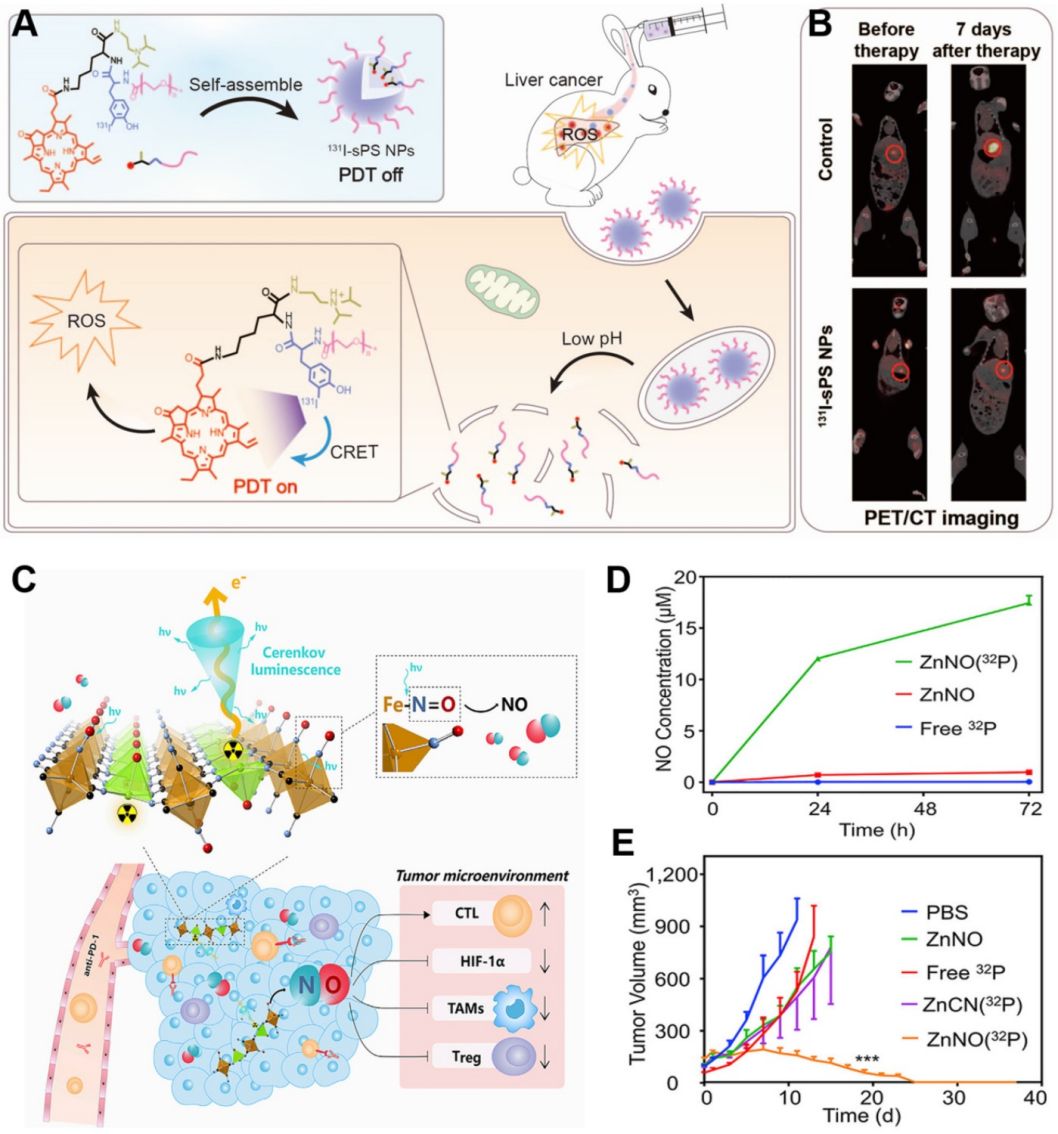
** (A)** Schematic of pH-activatable CRET-PDT. **(B)** Representative imaging of VX2 liver tumor-bearing rabbits using PET/CT before and at seven days after the indicated treatments. Reproduced with permission 96. Copyright 2021, Wiley-VCH. **(C)** Schematic of ^32^P-triggered NO release for enhanced radioisotope-immunotherapy. **(D)** Concentration of NO released from ZnNO(^32^P) nanosheets at various time points. **(E)** Growth curves of CT26 tumors in mice after various treatments. Reproduced with permission 99. Copyright 2019, Elsevier.

The low efficiency of CR limits its therapeutic efficacy 93, which combination therapy may improve 94. We have developed a ZGC nanoplatform conjugated to ^131^I-labeled ZnPc(COOH)_4_ (^131^I-ZGCs-ZnPcC4) that can support both radiotherapy as well as continuous, radiation-induced PDT through energy transfer from the ionizing radiation to ZGCs and then to ZnPc(COOH)_4_ 95. In another approach, we engineered pyropheophorbide-a containing PEG and a diisopropylamino group with an ^131^I-labeled tyrosine, which self-assembled into pH-sensitive NPs 96. These NPs exhibited minimal phototoxicity in normal tissue because of the quenched photodynamic effect, and under acidic conditions, they disassembled to promote the generation of ROS to achieve PDT and radiotherapy against deep-seated tumors (**Figure 12**A). Their strong accumulation in tumors makes the NPs effective against 4T1 tumors in mice and VX2 liver tumors in rabbits (**Figure 12**B). Thus, this strategy shows promise for deep tumor therapy. Combination therapy also has been developed in which the precursor of protoporphyrin IX called 5-aminolevulinic acid (ALA) and ^131^I are co-loaded into biomimetic exosomes. In the tumor microenvironment, where mitochondria are abundant, the ALA is converted to protoporphyrin IX, minimizing CRIT side effects on normal tissues 97.

Functionalized inorganic materials can also achieve multiple therapeutic effects. Titanium-oxo nanoclusters have been reported for CR-induced photo/chemodynamic therapy of tumors 98. CR can drive efficient production of hydroxyl radical (·OH) from titanium-oxo nanoclusters due to the enhanced separation of hole (h^+^)-electron (e^-^) pairs. The reaction of h^+^ and H_2_O provides type I PDT, while the transferred e^-^ augmented Ti^3+^ provides chemodynamic therapy. Besides, ^32^P-labeled single-layer 2D nanosheets have been constructed by mixing Zn^2+^ ions and sodium nitroprusside [Na_2_Fe(CN)_5_NO]. CR from ^32^P can persistently stimulate nanosheets to release NO, which can modulate the tumor microenvironment to induce anti-tumor immunotherapy and improve the efficacy of radionuclide therapy (**Figure 12**C,D) 99-100. This nanosystem has been extended to include immune-checkpoint blockade therapy against anti-programmed cell death protein 1 in order to achieve sustainably strong immune responses that effectively suppress tumor growth (**Figure 12**E).

## 6. Conclusion and perspectives

CR-activated probes take advantage of the ability of CR to pass through tissue without attenuation, enabling detection and treatment of deep-seated tumors. This review has focused on probes that can be activated by CR and that provide theranostics *via* CRET or production of reactive species/drug release. While these activatable probes can also be excited by ionizing radiation from radiotracers, especially lanthanide-based downconversion probes, the present review demonstrates the promise of approaches based on activation by CR. Further development of CRET will depend on the design of biocompatible optical probes with large Stokes shifts in the “deep NIR” to NIR-II regions. Future work should aim to overcome several limitations of CRIT, such as inefficient delivery of CR to therapeutic drugs in tumors, short duration of CR excitation, and damage to normal tissue during CRIT. In any case, suitable CR emitters and CR-responsive photosensitizers or drugs must always be chosen carefully in order to maximize tumor targeting and tumor cytotoxicity. Radionuclides with long half-lives are particularly promising as CR emitters, because they continuously generate CR to activate CR-responsive photosensitizers or drugs while also providing radiotherapy. Although CR remains in early stages of preclinical development, we believe that its use as an *in situ* light source will broaden the possibilities for imaging-based theranostics of cancer.

## Figures and Tables

**Table 1:** The properties of radionuclides used for CR applications 23, 24

Radionuclide	Half-life	Decay mode	Energy (keV)	Mean photo yield per disintegration	Clinical application
^18^F	109.8 min	β^+^ (97%)	633.5	1.32	PET
^64^Cu	12.7 h	β^+^ (17.8%)	653	0.557	PET
^68^Ga	67.6 min	β^+^ (88%)	1899	33.9	PET
^89^Zr	78.4 h	β^+^ (23%)	2400	2.29	PET
^124^I	4.17 d	β^+^ (11.7%)	1534	8.97	PET
^32^P	14.26 d	β^-^ (100%)	1710	28.1	Radiotherapy
^90^Y	64 h	β^-^ (99.9%)	2280	47.3	Radiotherapy
^131^I	8.02 d	β^-^ (89.9%)	606	0.669	Radiotherapy
		γ (81.7%)	364		
^177^Lu	6.73 d	β^-^ (78.6%)	498.3	0.141	Radiotherapy
^198^Au	2.69 d	β^-^ (98.9%)	960.7	Not reported	Radiotherapy

**Table 2:** Summary of CR-activated optical probes for imaging applications

Activatable probe	CR emitter	Combination way	CRET emission	Imaging subject	Ref.
Qtracker705	^64^Cu and ^18^F	Unbound	705nm	Subcutaneous pseudotumors	33
three CdSe/ZnS QDs	^131^I	Unbound	665nm, 705nm, 800nm	Subcutaneous pseudotumors	34
cRGD-QD605	^89^Zr-labeled deferoxamine-trastuzumab	Unbound	605nm	HER2/neu-expressing xenografts	35
CdSeTe/CdSe/CdZnS	^89^Zr	Extrinsic chelation	710nm	sentinel lymph nodes and prostate cancer	36
CdSe/ZnS	^64^Cu	Intrinsic radiolabelling	526nm, 580nm, 636nm	U87MG tumor	37
CuInS/ZnS	^64^Cu	Intrinsic radiolabelling	680nm	U87MG tumor	38
PdSe QDs	Megavoltage X-ray radiation	Unbound	1030nm	Subcutaneous pseudotumors	39
Ba_0.55_Y_0.3_F_2_:Eu^3+^	^18^F-FDG	Unbound	597nm, 615nm, 692nn	Subcutaneous pseudotumors	43
Y_2_O_2_S:Eu^3+^	^68^Ga	Unbound	660nm	Subcutaneous pseudotumors	44
Gd_2_O_3_:Eu^3+^	^18^F-FDG	Unbound	620nm, 700nm	4T1 tumor	45
Eu_2_O_3_	^18^F-FDG	Unbound	620nm, 700nm	Subcutaneous 4T1 tumor and orthotropic HCC tumor	46-49
Eu_2_O_3_	^89^Zr	Intrinsic radiolabelling	620nm, 700nm	Lymph node and CT26-tumor	50
Tb/Eu complexes	^18^F, ^89^Zr	Extrinsic chelation	490, 545nm/ 620nm	Phantoms	51
Tb(DO2Apic)-DUPA[Eu(DO2Aphen)-DUPA]	^18^F-FDG	Unbound	490, 545nm/ 620nm	PC3-PIP tumor (intratumoral treatment, IT)	52
Gold nanoclusters	^18^F-FDG	Unbound	680-700 nm	subcutaneous breast carcinomas	54
Gold nanoclusters	^64^Cu	Intrinsic radiolabelling	667nm	U87MG tumor	55
ZnGa_2_O_4_:Cr^3+^	^18^F-FDG	Unbound	695nm	4T1 tumor	61
Fluorescein sodium	^18^F-FDG, ^11^C-CHO	Unbound	520-530nm	subcutaneous 4T1 tumor and orthotropic HCC tumor	64
Fluorescein, rhodamine 6G, rhodamine 101, gresyl violet, cyanine 5, and indocyanine green	^90^YCl_3_	Unbound	710nm	EMT6-tumor (IT)	65
Phthalocyanine-pyranine conjugates	^18^F-FDG	Unbound	700nm	Subcutaneous pseudotumors	67
Naphthofluorescein derivatives	^18^F	Intrinsic radiolabelling	pH-sensitive	Normal mice	68
aluminum phthalocyanine, platinum II G4 (PtG4)	Megavoltage X-ray radiation	Unbound	780nm	Various tumor	69-72
[Ir(pq)_2_(bpy)]Cl liposome	^18^F-FDG	Unbound	570nm	4T1 tumor (IT)	74
Pluronic five different dyes-doped F127 silica nanoparticles	^32^P-ATP	Unbound	840nm	Phantoms	75

**Table 3:** Summary of CR-activated probes for therapeutic applications

Activatable probe	CR emitter	Combination way	CRIT	Treated subject	Ref.
TiO_2_-Tf-Tc	^18^F-FDG	Unbound	PDT	HT1080 tumor	79
TiO_2_-Tf	^89^Zr	Intrinsic radiolabelling	PDT	Multiple myeloma	80
Titanocene-loaded nanoparticles	^18^F-FDG	Unbound	PDT	Metastatic breast cancer and disseminated multiple myeloma	81
TiO_2_	^68^Ga-BSA	Unbound	PDT	4T1 tumor (IT)	82
Chlorin e6 loaded hollow mesoporous silica NPs	^89^Zr	Intrinsic radiolabelling	PDT	4T1 tumor (IT)	83
Porphyrin surface-modified magnetic NPs	^89^Zr	Intrinsic radiolabelling	PDT	4T1 tumor	84
Df-PPN	^89^Zr-labeled Df-PPN	Unbound	PDT	4T1 tumor	85
IRDye700DX	^18^F-FDG	Unbound	Photoimmunotherapy	A431-luc tumor	88
Folate-modified DOX/nanomicelles	Megavoltage X-ray radiation	Unbound	Chemotherapy	Hela tumor	92
ZGCs-ZnPcC4	^131^I	Extrinsic chelation	PDT-radiotherapy	4T1 tumor (IT)	95
sPS NPs	^131^I	Extrinsic chelation	PDT-radiotherapy	4T1 tumor-bearing mice and VX2 liver tumor-bearing rabbit	96
EM@ALA	^131^I	Extrinsic chelation	PDT-radiotherapy	4T1 tumor-bearing mice	97
Titanium-Oxo nanoclusters	^18^F-FDG	Unbound	Photo/chemodynamic therapy	HepG2 subcutaneous tumor	98
Single-layer 2D nanosheets	^32^P	Intrinsic radiolabelling	Radioisotope-immunotherapy	4T1 tumor (IT)	100
